# Vinegar-processed frankincense extracts alleviate colorectal cancer by butyric acid mediating M1 tumor-associated macrophage pyroptosis

**DOI:** 10.1186/s13020-025-01260-5

**Published:** 2025-12-01

**Authors:** Shitao Peng, Zhenli Liu, Zhiqian Song, Chun Wang, Zheng Yu, Ning Zhao, Wenjie Lu, Zhangchi Ning, Aiping Lyu

**Affiliations:** 1 Institute of Basic Theory for Chinese Medicine, China Academy of Chinese Medical Sciences, Beijing, 100700 China; 2 School of Chinese Medicine, Hong Kong Baptist University, Hongkong, 00825 China; 3 Institute of Basic Research in Clinical Medicine, China Academy of Chinese Medical Sciences, Beijing, 100700 China

**Keywords:** Frankincense, Vinegar-processed, Colorectal cancer, Butyric acid, M1 tumor-associated macrophage pyroptosis

## Abstract

**Background:**

*Olibanum* (RF), a traditional Chinese medicinal resin, shows efficacy in colorectal cancer (CRC) treatment. Its vinegar-processed form (PF) is clinically recognized for enhanced therapeutic effects, with prior mechanistic studies focusing on lipophilic components like boswellic acids. Yet, the regulatory mechanisms of PF's aqueous extracts remain unclear.

**Methods:**

The aqueous extracts of RF and PF were characterized and compared through transmission electron microscopy (TEM), nanoparticle analysis, and protein profiling. The accumulation of these fractions in feces was confirmed using DiR dye labeling. A mouse CRC model was employed to evaluate and compare the therapeutic effects of RF and PF. The composition of butyric acid-producing microbiota was analyzed using 16S rRNA gene sequencing and metagenomics. Butyric acid levels were quantified using ultra-high-performance liquid chromatography coupled with triple quadrupole mass spectrometry (UHPLC-TQ-MS). Macrophage phenotypes were assessed via flow cytometry, while mRNA and protein expression levels were determined through RT-qPCR and western blot analysis.

**Results:**

PF aqueous extracts exhibited distinct morphology, particle size, and protein content and had a superior therapeutic effect in alleviating CRC compared to RF. Further analysis confirmed that both RF and PF accumulated in feces and modulated the butyric acid metabolism of gut microbiota. The increased levels of butyric acid contributed to CRC alleviation by promoting the polarization of M1 tumor-associated macrophages (TAMs) and suppressing the pyroptosis of M1 TAMs.

**Conclusion:**

The study confirmed that vinegar-processed frankincense enhances its therapeutic effect on CRC by modulating M1 tumor-associated macrophages, which may provide efficient treatment of CRC from the perspective of host-gut metabolic interactions.

**Graphical Abstract:**

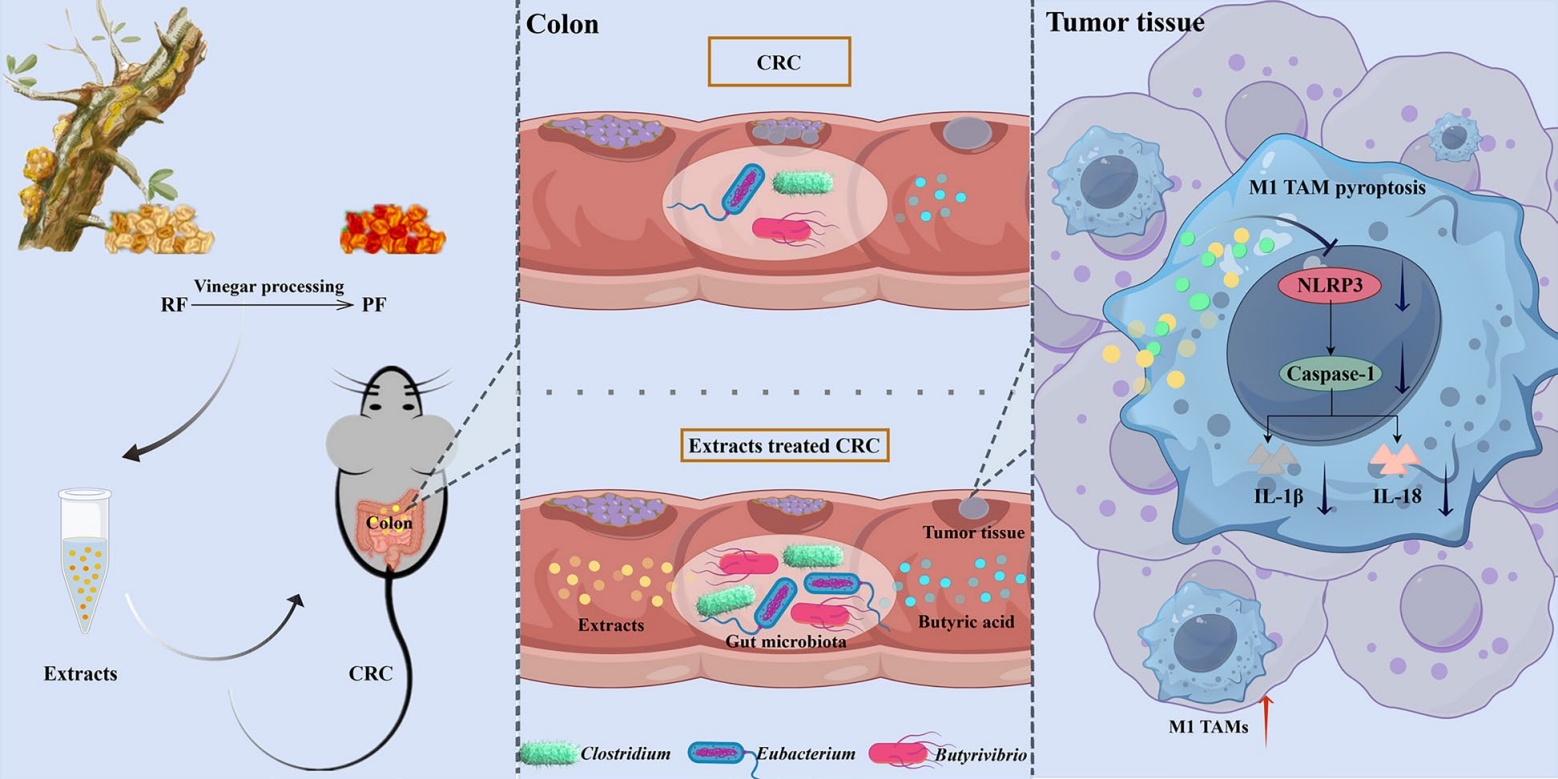

**Supplementary Information:**

The online version contains supplementary material available at 10.1186/s13020-025-01260-5.

## Introduction

*Olibanum* (known as frankincense) (RF), a resin exuded from the bark of *Boswellia carterii* Birdw, is commonly prescribed in both Indian and Chinese medicine for its therapeutic efficacy in cancer diseases [1, 2]. Based on the traditional theory of Chinese medicine, vinegar-processed frankincense (PF) served as a kind of formula applied in Chinese clinical practice and was believed to enhance the therapeutic effects. Modern pharmacological studies have further confirmed its significant efficacy in the treatment of inflammatory diseases and various cancers such as colorectal cancer (CRC) [1, 3].

Previous studies on the anti-cancer mechanism of frankincense have mostly focused on its lipid-soluble components, particularly the pentacyclic triterpenoid boswellic acids. These compounds exert direct anti-tumor effects by inhibiting tumor cell proliferation through blocking topoisomerase I activity, regulating the caspase-3/Bcl-2 pathway to induce apoptosis, and suppressing angiogenesis [4]. In addition, frankincense essential oil has been shown to exert selective cytotoxicity against CRC cells by disrupting mitochondrial function [3]. However, the water extract of frankincense, as an easily accessible component in clinical TCM preparations, has received far less attention, and its potential anti-CRC activity and underlying mechanism remain unclear.

Vinegar processing is a classic processing technique in TCM, which can enhance the efficacy of TCM and reduce its toxic and side effects [5]. For frankincense, PF is prepared through vinegar processing and is clinically used to strengthen its effect of "promoting blood circulation to relieve pain". Existing studies on PF have also mainly focused on optimizing its lipid-soluble components: vinegar can disrupt the crystal structure of frankincense resin to increase the solubility of boswellic acids [5], and improve its oral bioavailability by regulating bile acid metabolism [6]. Nevertheless, systematic research on the water extract of PF remains a gap—currently, it is unclear whether vinegar processing can alter the physicochemical properties of frankincense water extract, and whether these changes contribute to enhancing its anti-CRC efficacy. More importantly, no studies have explored whether the water extract of PF interacts with the gut microbiota to exert therapeutic effects.

CRC is the second most common cause of cancer-related deaths in developed nations [7]. According to the findings of multiple studies, there appears to be a consensus that gut microbiota can compromise the immune barrier and contribute to the progression of CRC particularly through the involvement of tumor-associated macrophages (TAMs). TAMs are the most abundant innate immune cells in the tumor microenvironment [8]. Based on functional differences, TAMs can be divided into two phenotypes: M1-type TAMs (marked by CD86, iNOS, and CD169), which secrete pro-inflammatory cytokines to enhance anti-tumor immunity; and M2-type TAMs (marked by CD206, CD163, and CD204), which promote tumor invasion and immune evasion [9, 10]. Pyroptosis is a pro-inflammatory programmed cell death mediated by the NLRP3 inflammasome and caspase-1, which can impair the anti-tumor function of M1-type TAMs: activated NLRP3 triggers the cleavage of caspase-1, leading to gasdermin D (GSDMD)-dependent membrane rupture and the release of interleukin (IL)-1β/IL-18, which ultimately resulting in a reduction in M1-type TAMs [11]. A growing body of evidence indicates that excessive pyroptosis of M1-type TAMs accelerates CRC progression [12, 13]; thus, the “NLRP3-caspase-1-IL-1β/IL-18” pathway has become a potential therapeutic target.

Butyric, a key short-chain fatty acid (SCFA) produced by the gut microbiota through the fermentation of dietary fiber, is a crucial mediator of crosstalk between the gut microbiota and immune cells [14]. Recent studies have shown that butyric can regulate the function of M1-type TAMs through multiple mechanisms: activating the GPR109A receptor to reduce mitochondrial reactive oxygen species production, thereby inhibiting NLRP3 inflammasome activation [15]; acting as a histone deacetylase inhibitor to suppress NLRP3 transcription and downregulate the expression of pro-inflammatory cytokines such as IL-1β [16], and promoting the polarization of M1-type TAMs by upregulating CD86 expression and enhancing TNF-α secretion [17]. In CRC patients, the abundance of butyric-producing bacteria and fecal butyric levels are significantly reduced [18, 19], which highlights the protective role of butyric in CRC. However, no studies have yet linked frankincense to the "gut microbiota-butyric-M1-type TAMs pyroptosis" axis, which is the key research gap that this study aims to fill.

Notably, previous studies on the anti-CRC effect of frankincense have mainly focused on the direct interaction between its lipid-soluble components and tumor cells [3]. In contrast, this study is the first to explore the role of the water extract of PF in regulating the gut microbiota-immune axis for CRC treatment. We propose the following hypothesis: Vinegar processing can alter the properties of frankincense water extract and enhance its accumulation in the colon; the accumulated water extract of PF then enriches butyric-producing gut microbiota and increases intestinal butyric levels; subsequently, butyric inhibits the NLRP3 inflammasome pathway, promotes the polarization of M1-type TAMs and suppresses their pyroptosis, ultimately exhibiting superior anti-CRC efficacy compared to RF water extract.

## Materials and methods

### Chemicals and materials

All standards of S/MCFAs were acquired from Shanghai Zhenzhun Co., Ltd. (Shanghai, China). The antibodies utilized in the western blot analysis included NLRP3 (Abcam, ab263899), IL-1*β* (Abcam, ab283818), IL-18 (Abcam, ab207323), Caspase-1 (Abcam, ab138483), and *β*-actin (beyotime, AF0003). All antibodies used for flow cytometry were procured from BioLegend Co., Ltd. (San Diego, CA, USA).

### Frankincense processing and aqueous extract preparation

RF was identified according to Chinese Pharmacopeia [20]. PF was prepared following a previous study [21]. After complete crushing, 2 g RF or PF powder was suspended in 12 mL of phosphate buffer saline (PBS), and the material was centrifuged (4 ℃, 5500 × g) for 20 min. The centrifugation procedure was repeated twice. The supernatant was ultracentrifuged (4 °C, 150,000 × g) for 2 h. Sucrose gradients of 8%, 30%, 45%, and 60% (g/v) were applied to the suspension before ultracentrifuging (4 ℃, 150,000 × g) for another 2 h. The bands between the layers of 30%/45% were harvested.

### Transmission electron microscopy (TEM) and nanoparticle tracking analysis

Aqueous extract samples were filtered through a 0.22 μm polyether sulfone syringe filter (Millipore, USA) to remove large aggregates before particle characterization. For TEM imaging, 50 μL of samples were applied to copper grids coated with formvar, followed by the addition of 50 μL of 2% uranyl acetate for 1 min, and subsequent drying of the samples at room temperature. The particle size and zeta potential of the aqueous extract fractions were determined using the Zeta Sizer 2000 (Malvern, UK).

### Protein components of RF and PF

0.05 mg/mL proteinase K (Beyotime, China), a broad-range endocytic protease, was used for RF and PF digestion. To separate the proteins, we selected 12% polyacrylamide gel for electrophoresis.

### Experimental animals

Four-week-old male C57BL/6 mice (18 ~ 22 g) were obtained from the National Institutes for Food and Drug Control and given a one-week acclimation period before the commencement of the experiments. Standard rodent food and water were provided to the mice. The study received approval from the Animal Experiment Ethics Committee on October 20th, 2021, with the registration number IBTCMCACMS21-2110–01. The laboratory’s rodent license (No. SYXK 2021–0017) was granted by the Ministry of Science and Technology in Beijing, China. The animal experiments were carried out following protocols approved by the Animal Ethics Committee of the Institute of Basic Theory of Chinese Medicine, China Academy of Chinese Medical Sciences.

### RF and PF labeling and distribution in vivo

DiR dye (Biorigin, China) was used to label RF and PF. Following a wash with PBS, the suspension of RF and PF was incubated at room temperature for 30 min in 10 mL of PBS containing 10 μM of DiR. The labeled RF and PF were orally administered to C57BL/6 mice at a dosage of 1.5 g/kg (6 mice per group). Mice were sacrificed and organs were collected at 6 h after administration. The Odyssey CLx Imaging System (Licor Biosciences) was utilized to visualize the labeled RF and PF in the mice's organs and feces.

### Establishment of CRC model and animal studies

C57BL/6 mice were initially administered an intraperitoneal injection of azoxymethane (AOM) (A5486, Sigma, USA) solution at a dosage of 10 mg/kg. After 5 days, mice were given three cycles of dextran sulfate sodium (DSS) (42,867, Sigma, USA) modified as described [22]. In the initial two cycles, mice were given 2% DSS for 5 days, followed by drinking water for 10 days. In the third cycle, mice were administered with 2% DSS for 5 days, followed by 5 days of water. Starting on day 10, the mice were randomly divided into eight groups of six each: the model group, oxaliplatin group, RF high dose group (RF-H) at 1.5 g/kg, RF medium dose group (RF-M) at 0.75 g/kg, RF low dose group (RF-L) at 0.38 g/kg, PF high dose group (PF-H) at 1.5 g/kg, PF medium dose group (PF-M) at 0.75 g/kg, and PF low dose group (PF-L) at 0.38 g/kg. Oxaliplatin was administered at a dosage of 2 mg/kg via injection. The control group of 6 mice was intraperitoneally injected with saline and fed with water without DSS during the entire experiment. The control group and model group were only given PBS. Throughout the experimental period, the body weight, stool characteristics, and stool bleeding of the mice were regularly monitored. The disease activity index (DAI) was calculated as the combined score of weight loss, stool characteristics, and bleeding. The scoring system was as follows: body weight (0, no loss; 1, 1–5% loss; 2, 5–10% loss; 3, 10–15% loss; and 4, > 15% loss); stool characteristics (0, normal; 2, loose stool; and 4, diarrhea); bleeding (0, no blood; 2, presence of blood; and 4, gross blood).

As part of the induction and maintenance procedure, mice were anesthetized using 3% isoflurane. The colon and fecal samples were then meticulously isolated and gathered. The spleen was measured, and the index of the spleen was calculated based on the following formula: spleen weight (mg)/ body weight (g). Occult/gross bleeding score evaluated colon damage on a scale of 0 (normal appearance) to 4 (gross bleeding). The colon's length was quantified. Then, the intestinal tract was longitudinally opened, and the number and size of the tumor were determined. The colon tissues underwent staining with hematoxylin and eosin for the observation of pathological changes.

### Chemical derivatization, sample preparation, and ultra-high performance liquid chromatography triple quadrupole mass spectrometry (UHPLC-TQ-MS) analysis for S/MCFAs

The derivatization parameters referenced by Garcia-Rivera et al. [23] were implemented in our lab. Briefly, 20 mg fecal samples were homogenated and dispersed in 200 μL of methanol which contains 50 μM internal standards (ISTDs). The homogenate substance was subsequently centrifuged, and 30 μL of the resulting supernatant was combined in a separate tube with 20 μL of 200 mM 3-Nitrophenylhydrazine (3NPH), 20 μL of 400 mM 1-(3-dimethylaminopropyl)-3-ethylcarbodiimide hydrochloride (EDC) with 6% pyridine, and then incubated at 30 ℃ for 60 min. After incubation, the sample was dried nitrogen. The remaining material was dissolved again in 100 μL of 75% (v/v) methanol: H_2_O for subsequent UHPLC-TQ-MS analysis.

The analyses were carried out in a UHPLC-TQ-MS (Agilent, Santa Clara, CA, USA). Briefly, a 2 μL sample was injected into an Agilent Poroshell 120 EC-C18 column (2.7 μm, 4.6 × 75 mm) at 30 ℃. Gradient elution mode was employed with a flow rate of 0.5 mL/min. The elution solvents consisted of 0.1% (v/v) formic acid in water (A) and 0.1% (v/v) formic acid in ACN (B). The elution gradient over 32 min was as follows: 0 to 14 min, 15% to 32% B; 14 to 17 min, 32% to 35% B; 17 to 25 min, 35% to 60% B; 25 to 27 min, 65% to 100% B; 27 to 32 min, 100% B, followed by subsequent steps for column cleaning and equilibration. Multiple reaction monitoring (MRM) methods were established using commercial standards in negative mode. Individually optimized tube lens voltage, collision energy, and fragment ions were determined for each compound (Table S1). Mass Hunter quantitative analysis software (Agilent Corp.) was used to conduct data processing.

### 16S rRNA gene sequencing

To characterize the microbial composition, total genomic DNA was isolated from fecal samples using a fecal DNA extraction kit (QIAGEN, USA) according to the manufacturer's protocol. The V3-V4 region of 16S rRNA genes of the samples was amplified using primers 338F (ACTCCTACGGGAGGCAGCAG) and 806R (GGACTACHVGGGTWTCTAAT). The polymerase chain reaction (PCR) was carried out on a Mastercycler Gradient (Eppendorf, Germany). The cycling conditions consisted of an initial denaturation at 95 ℃ for 5 min, followed by 28 cycles of denaturation at 95 ℃ for 45 s, annealing at 55 ℃ for 50 s, and extension at 72 ℃ for 45 s, with a final extension step at 72 ℃ for 10 min. The QIAquick@ Gel Extraction Kit (QIAGEN, USA) was used to purify PCR products. The libraries underwent sequencing on the Miseq platform at Allwegene Company (Beijing). QIIME software (Version 1.7.0) was used to analyze the dataset. The sequences were grouped into operational taxonomic units (OTUs) at a 97% similarity threshold to assess richness and diversity indices [24].

### Metagenomics

Genomic DNA from the entire sample was isolated using a fecal DNA extraction kit (QIAGEN, USA) and sheared to 300 bp with the Covaris ultrasonic crusher. Following ultrasonic DNA fragmentation, Illumina-compatible adapters were ligated for end-repair. The DNA sequencing libraries were subjected to deep sequencing using the Illumina Novaseq platform at Allwegene Technology Co., Ltd. in Beijing, China. Image analysis and base calling refer to the process of analyzing and interpreting the raw data from Illumina sequencing using the Illumina pipeline v2.6. The raw sequence reads were subjected to trimming with a quality score below 20 and a length of less than 50 base pairs. The resulting clean raw reads were subsequently assembled using Prodigal software to generate contigs for subsequent prediction and annotation [25]. Subsequently, the non-redundant gene set was generated using CD-HIT software [26].

### Colon tumor dissociation and flow cytometry

To prepare a single-cell suspension, the tumor tissues were dissociated into a cell suspension with complete RPMI 1640 medium containing 0.75 mg/mL collagenase A for 30 min at 37 ℃. The cells were strained with a 70 mm strainer before being centrifuged for 10 min at 300 g. The cell layer was collected and resuspended in 40% Percoll and layered above 80% Percoll to generate a Percoll gradient. Live-dead staining and Fc-blocking were performed on the single-cell suspensions for 15 min at 4 ℃ in PBS. Then cells were incubated with FITC-anti-CD45, PE/DazzleTM 594-anti-F4/80, PerCP/Cyanine 5.5-anti-CD11b and APC-anti-CD86 antibodies for 20 min at 4 ℃ in dark. A BD FACSCelesta (BD Biosciences, San Jose, CA, USA) was performed to analyze the cells after they were washed with PBS. The data underwent processing using FlowJo software (FlowJo LLC, Ashland, OR, USA).

### Real-time quantitative PCR

The colonic tumor tissues and THP-1-derived M1 TAMs were subjected to RNA isolation using TransZol Up reagent, following the manufacturer’s instructions. NanoDrop 2000C was used to measure RNA concentration. Bio-Rad CFX-96 Master cycler (California, USA) was employed for real-time quantitative PCR analysis on cDNA samples to determine mRNA levels. Table S2 contains the primer sequences. The target mRNA was normalized to its corresponding *β*-actin mRNA.

### Western blot

The protein was extracted by lysis buffer added with 1 mM phenylmethanesulfonyl fluoride (PMSF) to prevent degradation. The BCA protein assay kit was employed to determine the protein concentration. Following denaturation and separation via sodium dodecyl sulfate–polyacrylamide gel electrophoresis (SDS-PAGE), the protein samples were transferred onto polyvinylidene fluoride (PVDF) membranes. Subsequently, the membranes were blocked for 2 h using 5% milk powder and then subjected to overnight incubation at 4℃ with primary antibodies and 1 h incubation at room temperature with secondary antibodies.

### Cell culture and analysis

THP-1 cells (RRID: CVCL_0006) were obtained from ATCC (VA, USA), maintained in RPMI-1640 medium (Gibco, Grand Island, NY, USA) supplemented with 10% fetal bovine serum (FBS) and 0.05 mM *β*-mercaptoethanol, and incubated at 37℃ and 5% CO_2_. After incubation with phorbol myristate acetate (PMA) (100 ng/mL) for 48 h, THP-1 cells were induced to differentiate into THP-1-derived macrophages. To replicate the M1 TAMs, macrophages were indirectly co-cultured with DLD-1 and HCT116 cells using Transwell plates (0.4 μm pore size, Corning) for 5 days and induced by the addition of lipopolysaccharide (LPS) (100 ng/mL) and interferon-*γ* (IFN-*γ*) (20 ng/mL) for 24 h. To determine the RF and PF uptake by macrophages, cells (1 × 10^6^/well) were co-incubated with PKH67-labelled RF and PF (10 μg/mL) for 12 h. Then cells were fixed with 4% paraformaldehyde for 10 min. After the cells were washed with PBS, 4,6-Diamidino-2-Phenylindole (DAPI) was added, and the mixture was incubated for an additional 15 min. Finally, cells were imaged using an Olympus FV10i confocal microscope (Olympus, Japan). Then M1 TAMs were treated with butyric acid (257 μg/mL), RF (100 μg/mL), PF (100 μg/mL), or vehicle control, respectively for 24 h. PE-anti-CD86 antibodies were used for fluorescent staining. The cell supernatant was collected and the concentrations of TNF-*α*, IL-1*β*, and IL-6 were quantified using ELISA kits. To investigate the effect of butyric acid, RF, and PF on *CASPASE-1*, *IL-1β*, and *IL-18* under the inhibition and activation state of NOD-like receptor 3 (*NLRP3*). M1 TAMs were subjected to serum starvation for 24 h in 0.5% FBS, followed by seeding in six-well plates at a density of 1 × 10^6^ cells per well. Subsequently, the cells were treated with either the inhibitor (JC-171, 8 μM) or agonist (BMS-986299, 3 μM) and then exposed to butyric acid (257 μg/mL), RF (100 μg/mL), PF (100 μg/mL), or vehicle control for 24 h. The cells were then harvested at the specified time points.

### Establishment of nude mouse tumor transplantation model and animal studies

4 × 10^5^ DLD-1 and HCT116 cells were cultured in a cancer medium within a six-well plate for 36 h until the desired cell density was reached. Subsequently, the cancer cell medium was replaced with THP-1 medium and incubated for an additional 24 h. The transwell insert containing the THP-1 derived M0 macrophages was then transferred to the six-well plate where cancer cells were cultured, and the co-culture was incubated for 5 days. Then the macrophages were incubated with butyric acid (257 μg/mL), RF (100 μg/mL), PF (100 μg/mL) and followed by co-culturing with HCT116 cells for 24 h. Wash HCT116 cells three times with sterile PBS before implanting into nude mice. In the subcutaneous tumor inoculation study, 4-week-old BALB/c nude mice were subcutaneously inoculated with HCT116 cells, and the body weight and tumor growth were monitored. After 7 days, the mice were sacrificed, and tumor samples were excised, weighed, and subjected to flow cytometry for macrophage analysis.

### Statistical analysis

The data were presented as mean ± SD and analysed using GraphPad Prism 8.0 (San Diego, USA). For overall group comparisons, the Kruskal-Walli’s test was used, followed by Dunn's post-hoc test for pairwise comparisons. For high-dimensional data analyses including 16S rRNA sequencing, KEGG pathway enrichment, and cytokine panels, the Benjamini–Hochberg method was applied to correct for multiple comparisons and control the false discovery rate. All statistical results were confirmed with SPSS 22.0 (IBM SPSS, USA). A P-value (or adjusted P-value for multiple comparisons) of less than 0.05 was considered statistically significant.

## Results

### Isolation, purification, and characterization of water-soluble fractions of RF and PF

**Fig.1:**
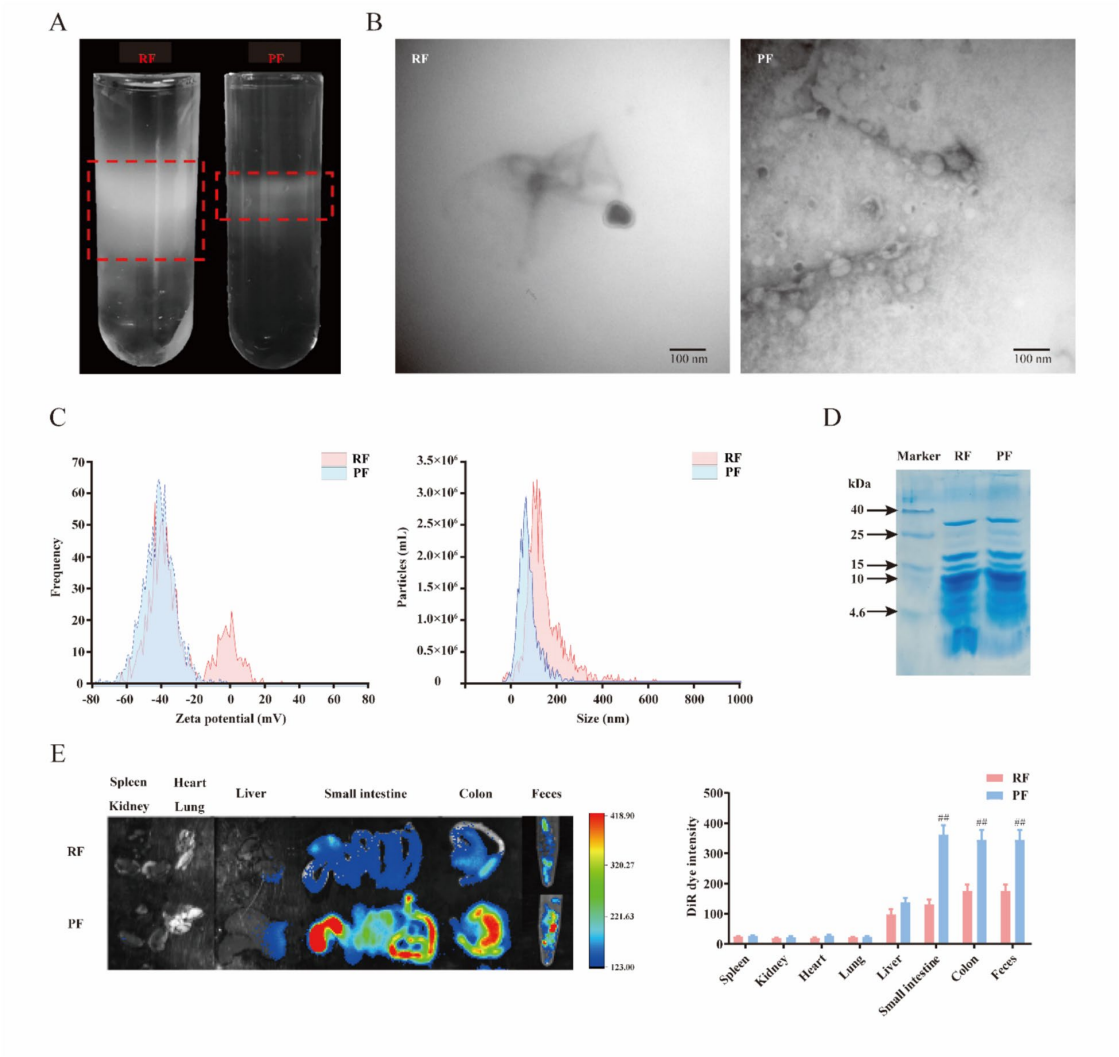
Characterization of water-soluble fractions in RF and PF. **A** RF and PF were isolated and purified using sucrose gradient ultracentrifugation. **B** The morphology of RF and PF is characterized by TEM. **C** Size distributions and zeta potentials of RF and PF. **D** The proteins in RF and PF were analyzed by 12% SDS polyacrylamide gel electrophoresis. **E** Representative image of the different organs and feces from mice receiving a gavage of DiR-labeled RF and PF

To isolate and purify the aqueous extract from RF and PF, the experiment was carried out by sucrose gradient ultracentrifugation according to the previous study [27]. As presented in Fig. 1A, the purified extracts of RF, and PF mainly accumulated at the 30/45% interfaces of the sucrose gradient. The results indicated that the purified production of PF was significantly decreased when compared with RF. Next, we investigated the characteristic change of extract from RF and PF. TEM is an effective tool for understanding morphology and size. There was a significant difference between RF and PF in their TEM images. PF displayed smaller particle sizes and were more abundant than RF (Fig. 1B). Zeta potential was used to reflect the surface charge of the extract. The results indicated that PF had an average negative zeta potential of − 41.2 mV, while that of RF was − 20.6 mV. This suggests that the stability of the PF may be enhanced, as smaller particles may be more likely to remain stable in vivo [28–30]. Consequently, this could result in a prolonged half-life and extended therapeutic efficacy. The average size of RF was 186 nm, and PF was 153 nm (Fig. 1C). The smaller particle size of PF may be advantageous for enhancing bioavailability, facilitating extracellular vesicle transit across cell membranes, and improving permeability through biological barriers such as the intestinal epithelium. To prove whether vinegar processing affects the protein compositions in the RF and PF, samples were analyzed on SDS-PAGE. It indicated that the proteins (< 4.6 kDa) in RF were decreased after vinegar processing (Fig. 1D). Furthermore, we evaluated the RF and PF uptake in vivo. The mice were administered DiR-labeled RF and PF, and analysis of the DiR positive in different organs and feces was shown in Fig. 1E. We found that most of the DiR-labelled RF and PF were in feces, colon, small intestine, and liver. However, few signals were detected in the lung, heart, kidney, and spleen. The mice treated with PF exhibited significantly stronger fluorescence intensity in the feces and colon compared with RF. The increased content of PF in the feces and colon might play a beneficial role in drug efficacy by regulating intestinal flora and gut immunity.

### RF and PF relieved the AOM/DSS-induced CRC in the mice model

**Fig. 2:**
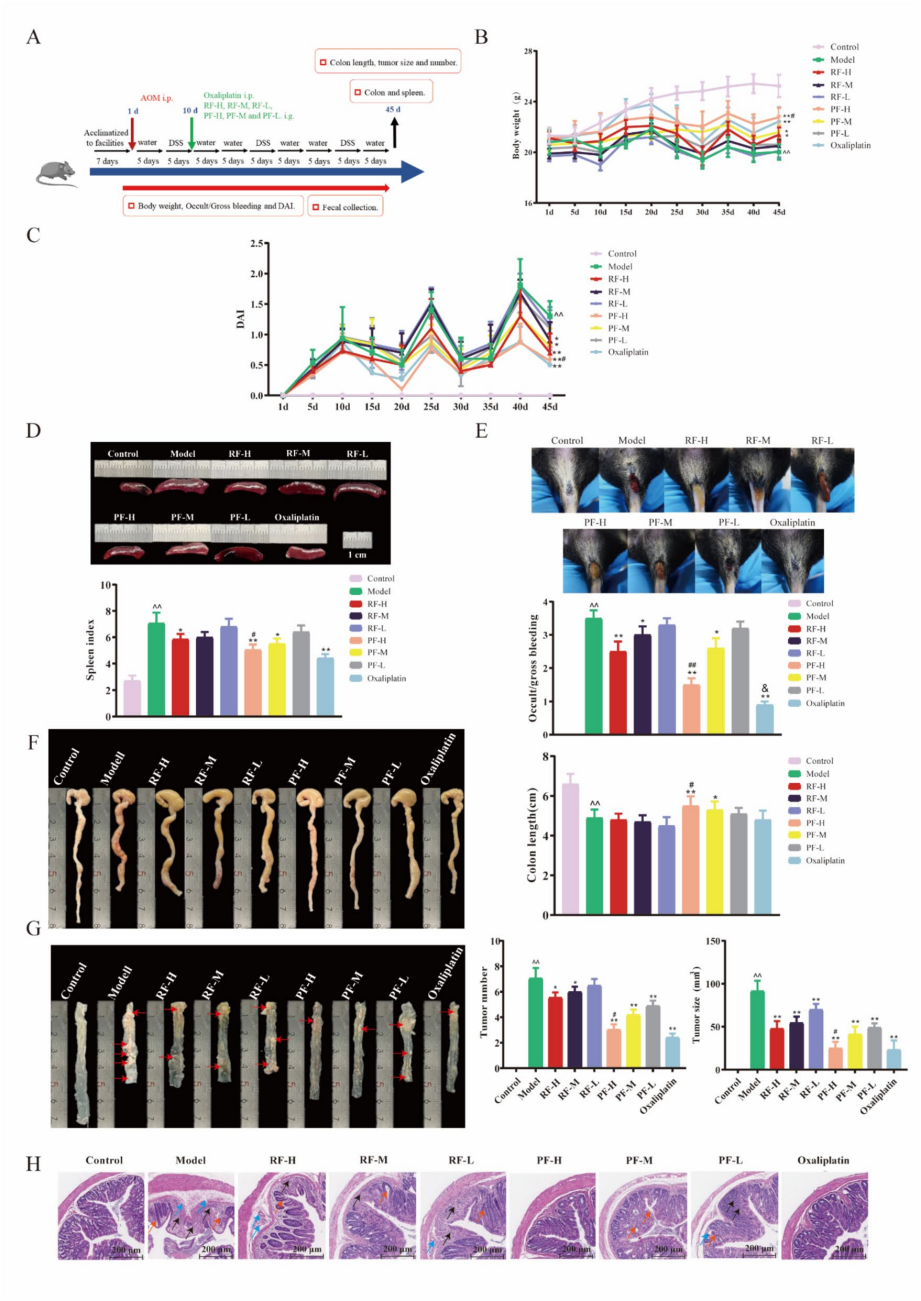
RF and PF treatment ameliorated AOM/DSS-induced CRC symptoms in mice. **A** Diagram of the experimental procedures. **B** The body weight recovery of CRC mice was facilitated by RF and PF. *n* = 6 individuals/group. **C** The DAI of CRC mice was decreased by RF and PF.* n* = 6 individuals/group. **D** PF decreased the spleen index of CRC mice.* n* = 6 individuals/group. **E** Treatment with RF and PF alleviated occult/gross bleeding of CRC mice.* n* = 6 individuals/group. **F** PF promoted the colon length recovery of CRC mice.* n* = 6 individuals/group. **G** PF decreased the tumor numbers and tumor sizes of CRC mice.* n* = 6 individuals/group. **H** HE staining of colon tissues. (The black arrow indicates inflammatory cell infiltration, the blue arrow indicates mucosal erosion, and the orange arrow indicates crypt destruction.)* n* = 6 individuals/group. Data differences were assessed using *Kruskal–Wallis* tests, respectively, ^^^*P* < 0.05 and ^^^^*P* < 0.01 versus the control group; ^*^P < 0.05 and ^**^P < 0.01 versus the model group; ^#^*P* < 0.05 and ^##^*P* < 0.01 versus the RF-H group; ^&^*P* < 0.05 versus the PF-H group

To investigate the pathological condition of CRC mice following RF and PF treatments, the study was carried out using in vivo models of AOM/DSS-induced mice, a well-established model for CRC. A schematic representation of the experimental protocol is presented in Fig. 2A. Throughout the treatment, the mice's body weights were monitored. The preservation of weight in the control group consistently increased, while other groups showed weight fluctuation. On the final day of administration (45 days), the body weight of the model group exhibited a significant reduction in comparison to that of the control group. RF and PF treatments improved body weight recovery of CRC mice, and PF-H showed the best elevation effect same as the positive drug oxaliplatin (Fig. 2B). The DAI was a comprehensive indicator based on animal weight, stool characteristic, bloody stool, and it was used to assess the progress of CRC. During the AOM/DSS-treated timescales of the experiment, DAI exhibited a robust increasing trend. The CRC mice performed higher DAI scores than those of RF and PF-treated mice. The most obvious decline was observed in the PF-H group (Fig. 2C). The spleen index was a preliminary indicator of immune function. As shown in Fig. 2D, model mice exhibited a much higher spleen index with a value was 7.2, while the value for the PF-H group was 5.3. PF-treated mice exhibited more mild gross bleeding when compared with CRC mice, and oxaliplatin showed the best effect (Fig. 2E). The average colon length shortening in the model mice was significantly prevented after PF-H treatments (Fig. 2F). To validate the efficiency of RF and PF, the number and size of tumors was determined. The results indicated that the tumor number and size in the model group were greatly increased, whereas RF and PF-treated groups were significantly suppressed, and PF-H performed a superior effect (Fig. 2G). In addition, it was found that AOM/DSS induced extensive colonic tissue damage, including robust inflammatory cell infiltration, mucosal erosion, and crypt destruction. RF and PF reduce inflammation and mucosal and crypt damage in the colon, particularly in mice treated with high doses of PF (Fig. 2H). These results suggest that RF-H and PF-H exhibited ameliorative influence on CRC mice, and PF-H showed superior efficacy than RF-H. High doses of RF and PF were selected to conduct the following experiments.

### RF and PF remodel the gut microbiota and elevate the butyric acid level of CRC mice

**Fig. 3:**
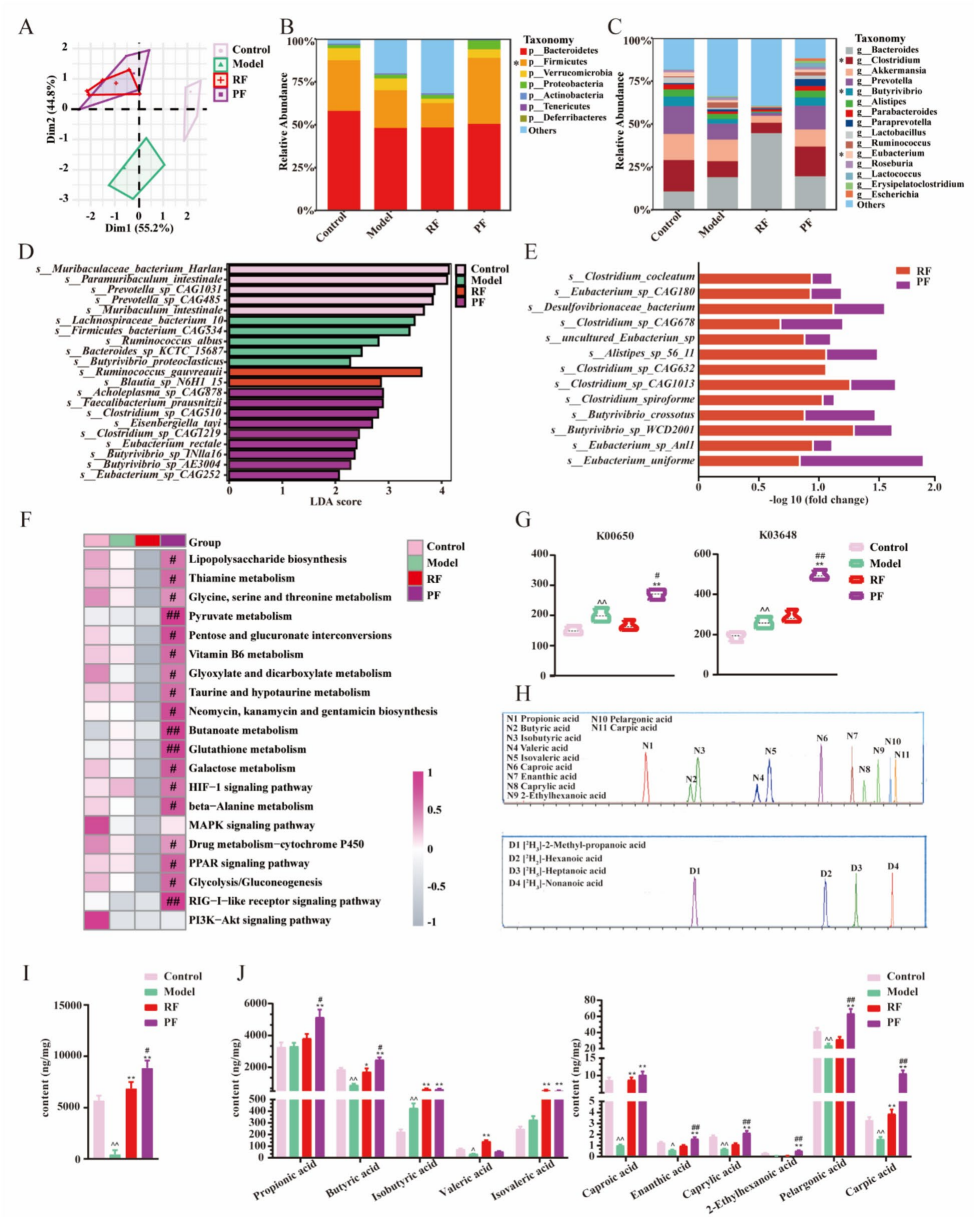
RF and PF regulated the S/MCFAs and gut microbiota in CRC mice feces. **A** PCA of gut microbiota in four groups.* n* = 6 individuals/group. **B** Mean relative abundance of gut microbiota at the phylum level. *n* = 6 individuals/group. **C** Mean relative abundance of gut microbiota at the genus level.* n* = 6 individuals/group. **D** LDA score was performed to compare enriched species in each group. The bar plot listed the significantly differential species (score > 2.0) in the C, M, RF, and PF groups.* n* = 6 individuals/group. **E** Fold change of the species related to butyric-produce in the model group with the most abundance.* n* = 6 individuals/group. **F** Heatmap analysis of KEGG level 3 pathway involved in butyric metabolism and immune response*. n* = 6 individuals/group. **G** PF enhanced the abundance of butanoate metabolism (K00650) and primary immunodeficiency (K03648).* n* = 6 individuals/group. **H** The LC–MS chromatography of eleven S/MCFAs in MRM mode. **I** RF and PF upregulated the total S/MCFAs levels in the feces of CRC mice.* n* = 6 individuals/group. **J** PF significantly increased the content of butyric acid, heptanoic acid, octanoic acid, nonanoic acid, and decanoic acid in the feces of CRC mice.* n* = 6 individuals/group. Data differences were assessed using *Kruskal–Wallis* tests, respectively, ^^^*P* < 0.05 and ^^^^*P* < 0.01 versus the control group; ^*^P < 0.05 and ^**^P < 0.01 versus the model group; ^#^*P* < 0.05 and ^##^*P* < 0.01 versus the RF group

As the accumulation of extract in the fecal of mice, we suspected that oral administration of aqueous extract affected the composition and functional potential. To explore the variation of gut bacteria, the microbiota sequences were analyzed. Rarefaction, shannon–wiener, rank-abundance, and species accumulation curves were conducted for the tested sample, indicating that the sequencing depth was sufficient (Fig. S1). Principal component analysis (PCA) was employed to visually illustrate the differences among the treatments. The findings demonstrated a distinct separation between control mice and model mice, which was also observed following the administration of RF and PF (Fig. 3A). The sequencing results provided insights into the composition of microbiota at various taxonomic levels. Specifically, at the phylum level, the relative abundance of firmicutes was significantly reduced in the model group but substantially increased following treatment with PF (Fig. 3B). Furthermore, at the genus level, DSS administration led to a notable decrease in *Clostridium*, a butyric-producing bacteria, which was effectively restored by PF treatment. Other butyric-producing bacteria such as *Eubacterium* and *Butyrivibrio* were also greatly increased by PF (Fig. 3C). Linear discriminant analysis (LDA) was employed to further clarify the distinct correspondence between gut microbiota and various groups. It was found that the species include *Clostridium_sp_CAG510, Clostridium_sp_CAG1219, Butyrivibrio_sp_INlla16, Butyrivibrio_sp_AE3004,* and *Eubacterium_sp_CAG252,* from *Clostridium, Eubacterium,* and *Butyrivibrio* genus was enriched by PF (Fig. 3D). The profile of butyric-producing species such as *Eubacterium_uniforme, Butyrivibrio_crossotus,* and *Clostridium_sp_CAG678* was significantly increased in both RF and PF groups compared with the model group, and they were markedly elevated by PF (Fig. 3E). The investigation of microbiota function is of paramount importance in comprehending the intricate connection between intestinal flora and CRC disease. Through the utilization of KEGG pathway analysis, we observed a noteworthy association between the bacteria in the PF group and inflammatory and immune pathways. Particularly, several of these pathways exhibited close ties to butyric metabolism and immune response, including the butanoate metabolism signaling pathway, pyruvate metabolism signaling pathway, PPAR signaling pathway, and RIG-I-like receptor signaling pathway (Fig. 3F). Furthermore, the abundance of butanoate metabolism (K00650) was significantly higher in the PF group compared to the RF group, and an upregulated trend was also observed for primary immunodeficiency (K03648) (Fig. 3G). These results indicate that butyric acid metabolism in gut microbiota may modulate the immune response associated with colitis. Next, UHPLC-TQ-MS was used to determine the levels of bacteria metabolites, short/medium chain fatty acids (S/MCFAs), in feces to confirm the discovery of the alteration of gut microbiota (Fig. 3H). It was found that the DSS caused lower fecal total S/MCFAs content. Both RF and PF showed elevated levels of total S/MCFAs, and PF revealed a more significant effect than RF (Fig. 3I). The contents of 11 kinds of S/MCFAs in feces were measured individually, and we noticed that PF showed a significant regulatory effect on S/MCFAs levels. Remarkably, butyric acid made up a large proportion of adjusted S/MCFAs (Fig. 3J). It implies a potential distinctive function of butyric acid in gut immunity.

### M1 TAMs pyroptosis of colon tumor was decreased by PF

**Fig. 4:**
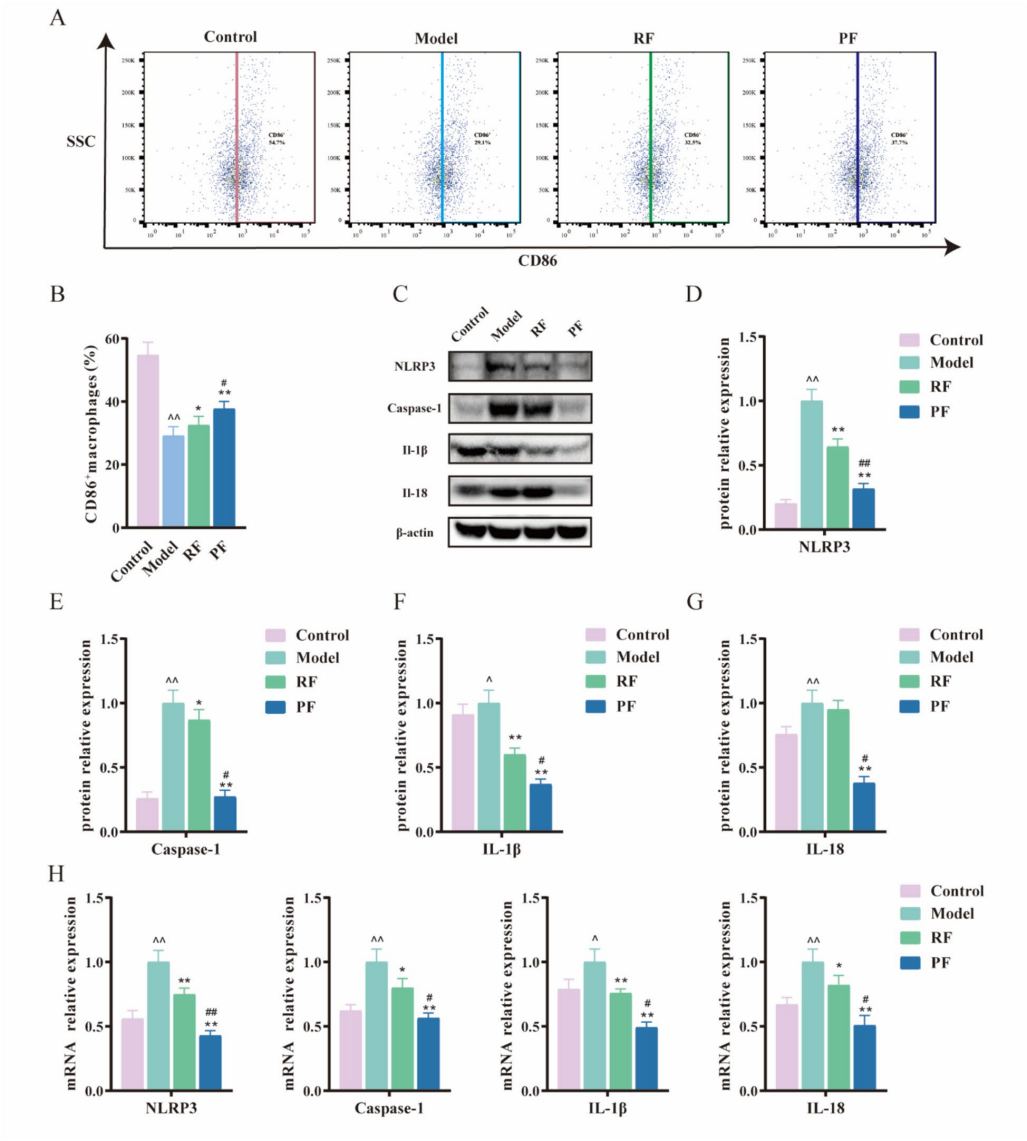
PF decreased M1 TAMs pyroptosis in mice colon tumor. **A** Representative flow cytometric plots of CD86^+^ macrophages. **B** Quantitative analysis of CD86^+^ macrophages. **C** Representative pictures of western blot: Nlrp3, Caspase-1, IL-1*β*, and IL-18. **D** Relative protein expression of Nlrp3 in mice colon tumor, *n* = 3. **E** Relative protein expression of Caspase-1 in mice colon tumor, *n* = 3. **F** Relative protein expression of IL-1*β* in mice colon tumor, *n* = 3. **G** Relative protein expression of IL-18 in mice colon tumor, *n* = 3. **H** Relative mRNA expression of Nlrp3, Caspase-1, IL-1*β* and IL-18 in mice colon tumor, *n* = 3. Data differences were assessed using *Kruskal–Wallis* tests, respectively, ^^^*P* < 0.05 and ^^^^*P* < 0.01 versus the control group; ^*^P < 0.05 and ^**^P < 0.01 versus the model group; ^#^*P* < 0.05 and ^##^*P* < 0.01 versus the RF group

TAMs, as the primary innate immune cells in the colon tumor tissues, are essential for maintaining intestinal homeostasis and initiating immune responses against intestinal disease. To investigate the functional mechanisms by which macrophages contribute to the alleviation of CRC after the administration of RF and PF, a flow cytometric analysis of macrophages in the colon tumor was conducted. The gating strategy employed FSC/SSC to identify live cells, followed by the identification of monocytes through CD45^+^. Subsequently, the expression of CD11b^+^ and F4/80^+^ was utilized to distinguish macrophages. The polarization of TAMs into M1 phenotypes was determined based on the presence of CD86^+^. The results indicated that the number of M1 TAMs was significantly decreased in the model group, while it showed a trend of recovery when treated with RF and PF (Fig. 4A, B). We also found that PF significantly increased the proportion of M1 TAMs in the colon. Furthermore, pyroptosis has also been highlighted in M1 TAMs polarization. Pyroptosis, a distinct form of proinflammatory cell death, relies on caspase-1 and is triggered by inflammasomes such as NLRP3. This activation induces cellular enlargement and membrane rupture, ultimately leading to the liberation of mature IL-1*β* and IL-18 from the cells. Reports show that diterpenes and triterpenes in frankincense have the potential to relieve cellular pyroptosis. To compare the effects of extract from frankincense with these compounds, we investigated the impact on these cytokines, and the protein expression of Nlrp3 was measured. An increased expression of Nlrp3 was observed in the model group. The expression of Nlrp3 in the PF group decreased when compared with RF (Fig. 4C, D). The protein expression levels of Caspase-1, IL-1*β*, and IL-18 were subsequently confirmed. Specifically, Caspase-1, IL-1*β*, and IL-18 proteins increase expression in the model group while tending to decrease expression in the RF and PF group. In addition, PF exhibited better regulation than RF (Fig. 4E-G). The mRNA expressions of Nlrp3, Caspase-1, IL-1 β, and IL-18 showed a consistent trend with protein expression (Fig. 4H).

### Butyric acid mediates the pyroptosis of M1 TAMs by inhibiting the NLRP3 signal pathway

**Fig. 5:**
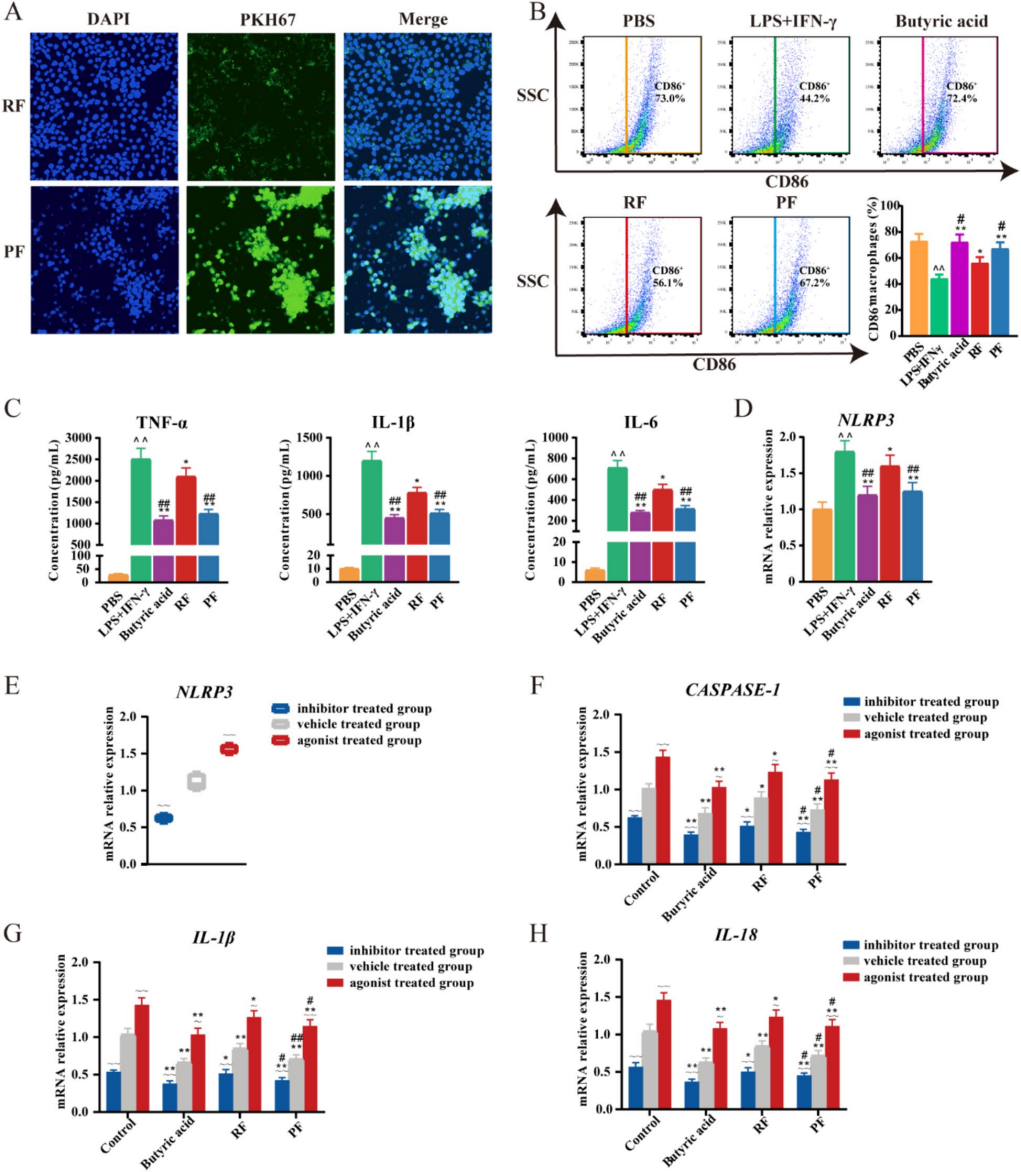
Inhibition of NLRP3 by PF reduces pyroptosis of M1 TAMs. **A** Confocal image of TAMs internalizing PKH67-labeled RF and PF extracts. **B** Flow cytometry detection of CD86^+^ expression on THP-1 derived M1 TAMs.* n* = 6 individuals/group. **C** Detection of the concentration of inflammatory cytokines (TNF-*α*, IL-1*β*, and IL-6) in the supernatant of THP-1 derived M1 TAMs.* n* = 6 individuals/group. **D** Relative mRNA expression of *NLRP3* in THP-1 derived M1 TAMs. *n* = 6 individuals/group. **E** Relative mRNA expression of *NLRP3* treated with inhibitor and agonist in THP-1 derived M1 TAMs. *n* = 6 individuals/group. **F** The impact on the modulation of *CASPASE-1* in THP-1 derived M1 TAMs. *n* = 6 individuals/group. **G** The impact on the modulation of *IL-1β* in THP-1 derived M1 TAMs. *n* = 6 individuals/group. **H** The impact on the modulation of *IL-18* in THP-1 derived M1 TAMs. *n* = 6 individuals/group. Data differences were assessed using Kruskal–Wallis tests, respectively, ^^^*P* < 0.05 and ^^^^*P* < 0.01 versus the PBS or control group; ^*^P < 0.05 and ^**^P < 0.01 versus the LPS + IFN-*γ* group; ^#^*P* < 0.05 and ^##^*P* < 0.01 versus the RF group; ^~^*P* < 0.05 and ^~~^*P* < 0.01 versus the vehicle-treated group

To further evaluate the uptake of RF and PF by TAMs, we labeled these extracts with PKH67 and utilized confocal microscopy to measure fluorescence intensity (Fig. 5A). Our findings suggest that both RF and PF groups exhibited PKH67 fluorescence, with PF demonstrating a significantly greater enhancement in extract uptake compared to RF. This suggests that TAMs phagocytosis predominantly mediates the internalization of these extracts and that PF exhibits a significantly superior effect compared to RF. Based on our previous findings, it was observed that PF exerted promotional effects on the activation of M1 TAMs and attenuated colon tumor pyroptosis by downregulating the NLRP3 signaling pathway. Subsequent evidence has demonstrated the inhibitory properties of butyric acid on NLRP3 [15, 16]. Consequently, we hypothesized that the potential ameliorative effects of PF on CRC may be attributed to their ability to enhance the content of butyric acid in the colon, thereby inhibiting NLRP3-mediated M1 TAMs pyroptosis. To confirm our hypotheses, THP-1-derived macrophages were used for the following study on the PF treatment mechanism. LPS and IFN-*γ* induced M1 TAMs were co-cultured with butyric acid, RF, and PF, and cells were collected for flow cytometric analysis. The results suggest that butyric acid, RF, and PF enhanced M1 TAMs polarization. A more obvious ascension was observed in the butyric acid and PF group (Fig. 5B). Correspondingly, the M1 TAMs markers, including TNF-*α*, IL-1*β*, and IL-6 were significantly downregulated by butyric acid and PF (Fig. 5C). In addition, *NLRP3* mRNA expression was detected, the results suggested that butyric acid and PF may decrease M1 TAMs pyroptosis by reducing *NLRP3* expression (Fig. 5D). To validate these hypotheses, THP-1 derived M1 TAMs were subjected to treatment with agonists and inhibitors. BMS-986299, a well-characterized *NLRP3* agonist, increased the mRNA expression level of *NLRP3*. Stimulation of *NLRP3* resulted in elevated expression levels of *CASPASE-1*, *IL-1β*, and *IL-18*. As shown in Fig. 5E-H, the upregulation of those genes was significantly alleviated by butyric acid. Conversely, JC-171 acted as an inhibitor of *NLRP3*, resulting in reduced expression of *CASPASE-1*, *IL-1β*, and *IL-18*. These results demonstrated that butyric acid was the reason for the adjustment of the NLRP3 signal pathway. Collectively, these data directly indicate that butyric acid and PF-mediated NLRP3 activation triggered pyroptosis in M1 TAMs.

### Butyric acid-intervened TAMs suppress tumor growth of CRC nude mice

**Fig. 6:**
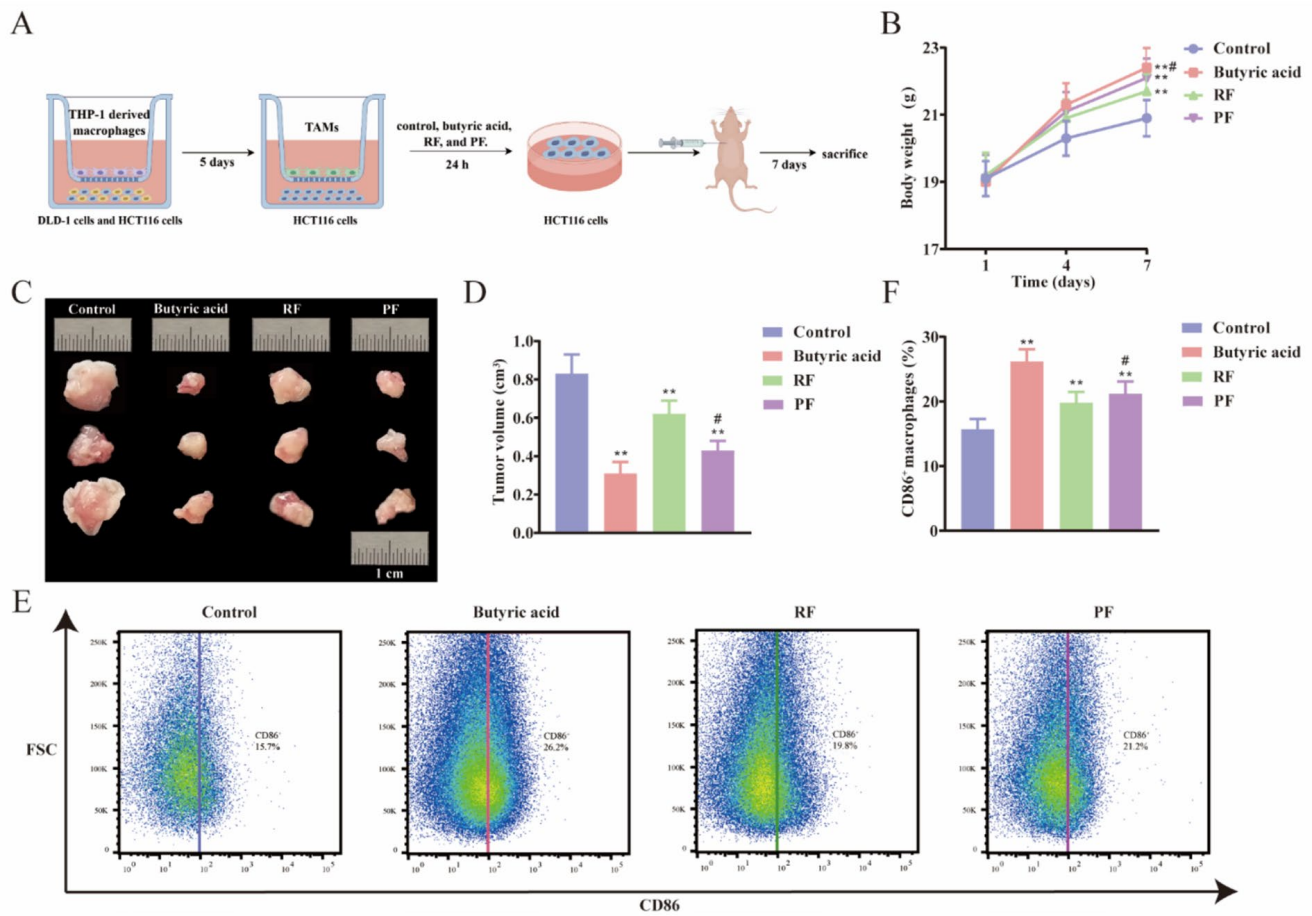
Subcutaneous tumors in nude mice were suppressed by butyric acid-intervened M1 TAMs. **A** Experimental flow chart of the nude mice study. **B** The restoration of body weight in nude mice was enhanced by RF and PF.* n* = 6 individuals/group. **C** Representative pictures of tumors after different treatments. **D** Quantitative analysis of the size of subcutaneous tumors. *n* = 6 individuals/group. **E** Representative flow cytometric plots of each group. **F** Quantitative analysis of the CD86^+^ macrophages.* n* = 6 individuals/group. Data differences were assessed using *Kruskal–Wallis* tests, respectively, ^^^*P* < 0.05 and ^^^^*P* < 0.01 versus the control group; ^*^P < 0.05 and ^**^P < 0.01 versus the model group; ^#^*P* < 0.05 and ^##^*P* < 0.01 versus the RF group

The therapeutic potential of butyric, RF, and PF for CRC was further investigated in vivo. To validate the alleviating effect of butyric on CRC, we employed subcutaneous tumor xenografts using nude mice injected with butyric, RF, and PF treated HCT116 cells to observe the effects on tumor growth. The entire experimental process is schematically illustrated in Fig. 6A. Notable changes in body weight were observed among the groups (Fig. 6B). Specifically, mice in the butyric and PF treatment groups exhibited higher body weights compared to those in the control group and RF group, indicating a potential lessening of the systemic effects of CRC and an improvement in overall health status. Furthermore, the results demonstrated significant suppression of tumor volume in the butyric and PF treatment groups. Tumor measurements indicated a reduction of approximately 50% in the butyric-treated group and 25% in the PF-treated group compared to the control group (Fig. 6C, D). This marked decrease underscores the potent anti-tumor efficacy of butyric acid and PF in inhibiting CRC progression. Flow cytometry analysis was conducted to evaluate the immune landscape within the tumor microenvironment (Fig. 6E, F). The proportion of M1 TAMs in tumor tissues was considerably higher in the butyric acid and PF treatment groups compared to the control group. The results indicated that these treatments may be modulating the immune status of the tumor microenvironment, potentially contributing to the observed anti-tumor effects. In conclusion, our study provides evidence that butyric acid and PF not only suppress tumor growth in CRC nude mice but also modulates the immune microenvironment by increasing M1 TAMs. These findings highlight the promising potential of butyric acid and PF as therapeutic agents in the treatment of CRC.

## Discussion

Traditionally, frankincense was applied in Chinese medicine for its multiple effects on inflammatory and cancer diseases in past decades. Vinegar processing as a traditional pharmaceutical method of Chinese herbal medicine, is believed to enhance the therapeutic effects of herbs, which was applied to frankincense when it was prescribed [20]. Research indicated that the lipid-soluble compounds, pentacyclic triterpene acids, serve as the main constituents in the disease's treatment [31]. However, there is a scarcity of published research on the comparative effects of aqueous extracts from RF and PF on CRC, as well as the mechanisms underlying the impact of processing on these extracts. Processing often enhances efficacy by altering the physical properties of herbs. For example, after vinegar processing of Corydalis Rhizoma, its alkaloids combine with acetic acid to form salts, increasing solubility in water and thereby enhancing analgesic effects [32]. In this paper, we investigated that aqueous extracts of Boswellia serrata have shown pronounced efficacy in alleviating CRC symptoms. Worthy of attention is that PF was observed to demonstrate a more pronounced effect in achieving CRC remission. PF’s smaller particle size may enhance penetration of the colonic mucus layer [33], increasing M1 macrophage uptake. Its more negative zeta potential reduces aggregation [6], ensuring uniform distribution and consistent interaction with immune cells. These physical changes may directly amplify immune modulation by improving cellular targeting.

The clinical application of frankincense has been hindered by the limited oral bioavailability of its lipid-soluble compounds [34]. In the present study, aqueous extracts of RF and PF were shown to accumulate in the intestine, potentially exerting therapeutic effects through interactions with the gut microbiota. The gut microbiota has been demonstrated to significantly influence drug efficacy by modulating both the pharmacokinetics and pharmacodynamics of drugs and through the production of bacterially derived metabolites [35]. Recent research has demonstrated that plant-derived extract can interact with the gut microbiota and stimulate the proliferation of advantageous bacteria, namely *Lactobacillus* and *Bifidobacterium*, while concurrently impeding the growth of detrimental bacteria, such as *Escherichia coli* and *Staphylococcus aureus* [36]. Butyric, a type of S/MCFA, is generated by the gut microbiota via the fermentation of dietary fiber. Numerous studies have revealed the anti-inflammatory, immunomodulatory, and anti-tumor properties of butyric, highlighting its pivotal role in sustaining intestinal homeostasis [37]. Butyric-producing bacteria, such as *Faecalibacterium prausnitzii*, *Roseburia spp.*, and *Eubacterium rectale* were confirmed to reduce in patients with inflammatory bowel disease (IBD) and CRC, suggesting a potential role in the pathogenesis of these diseases [18]. Research on microbiota-immune crosstalk has emerged as a hotspot in both TCM and oncology fields. For instance, Astragalus polysaccharides can regulate the gut microbiota, increase *Bifidobacterium* abundance, enhance macrophage activity, and strengthen anti-tumor immunity [38]. In CRC, gut microbiota dysbiosis leads to immune microenvironment disorder and reduced butyrate-producing bacteria, which impairs immune cell function and promotes tumor progression [8]. The regulation of the microbiota-butyric acid-immune axis by PF in this study aligns with this emerging research direction. We found that aqueous extract of frankincense was accumulated in the mice's colon and feces, which may indicate the influence of extract on gut microbiota. Moreover, our research further substantiates the significance of butyric acid-associated bacteria in the mechanism of mitigating CRC by PF.

UPLC-TQ-MS quantitative analysis showed that the content of triterpenoids in the aqueous PF extract was much lower than that in lipophilic extracts [5], indicating low extraction efficiency of triterpenoids by aqueous extraction. However, unique peptides identified in the aqueous PF extract may be key bioactive small molecules responsible for its efficacy [34]. These peptides may directly bind to targets such as NLRP3, participate in regulating macrophage pyroptosis and immune response, and thereby influence CRC progression. Intestinal immunity plays a critical role in the development and progression of CRC [39]. Within the intestinal microenvironment, macrophages are key immune cells that exhibit diverse functions and phenotypes [40, 41]. The pyroptosis of intestinal macrophages, particularly M1 TAMs, has garnered significant attention due to its potential impact on CRC progression and the anti-tumor immune response. The pyroptosis of M1 TAMs in the intestinal microenvironment of CRC can have profound functional consequences. It may lead to impaired anti-tumor immune responses, enhanced tumor growth and invasion, and the promotion of an immunosuppressive microenvironment [42, 43]. Thus, inhibition of TAM pyroptosis may exert a favorable impact on the progression of CRC. Our findings validate a comparable trend, demonstrating that PF exerts a mitigating influence on CRC through the inhibition of M1 TAMs pyroptosis.

We hypothesize that PF regulates M1 pyroptosis through a dual mechanism involving "direct action" and “indirect mediation”. Directly, vinegar processing alters the physical properties of PF, making it more easily phagocytosed by M1 macrophages. The bioactive small molecules contained in PF may directly bind to NLRP3 protein, inhibiting its oligomerization and inflammasome assembly, thereby reducing NLRP3 expression in M1 macrophages. Indirectly, PF modulates the gut microbiota, significantly increasing the abundance of butyrate-producing bacteria and elevating fecal butyrate levels. Butyric acid can inhibit histone deacetylation in the NLRP3 gene promoter region, reducing its transcription [16]; simultaneously, it activates the GPR109A receptor, reduces mitochondrial ROS production, and blocks NLRP3 activation signaling [15]. Previous studies have shown that frankincense itself does not contain butyric acid [5], and its efficacy depends on microbial transformation. In the tumor xenograft model, PF was less effective than butyrate, mainly possibly because exogenous butyrate can act directly on the tumor microenvironment, whereas PF requires gut microbiota fermentation to produce butyrate.

In this paper, our findings emphasize the therapeutic potential of RF, particularly those processed with vinegar, in modulating the gut microbiota and immune response, offering an effective and safe approach to CRC treatment. PF exhibits favorable clinical applicability. As a core component of classic TCM formulas, it undergoes metabolism by the gut microbiota in vivo and targets the intestinal microenvironment, which aligns with the pathogenesis of CRC. Acute toxicity studies showed that mice administered with frankincense at doses higher than 5 g/kg exhibited no mortality or organ damage [44, 45] suggesting high safety for clinical application, though further clinical research is still needed for verification. Manipulating macrophage viability and activity via pharmacological or immunological interventions may provide avenues for treatment. The specific molecular pathways and signaling networks involved in macrophage pyroptosis are needed. Subsequent studies plan to incorporate human CRC organoids and clinical tumor tissue samples to comprehensively evaluate the effects of PF on pyroptosis of human-derived macrophages, gut microbiota, and the immune microenvironment, thereby enhancing the clinical translational value of the research findings. Meanwhile, techniques such as Western blotting will be used to detect key downstream pyroptosis markers (e.g., GSDMD cleavage), to clarify the complete molecular pathway by which PF inhibits pyroptosis of M1 macrophages.

## Conclusion

The primary objective of the present investigation was to elucidate the mechanism by which aqueous extract of RF and PF ameliorate CRC. The research findings demonstrated that vinegar processing can alter the zeta potential, reduce the particle size, and decrease the levels of low molecular weight proteins in the extract. PF is involved in the modulation of gut microbiota butyric acid metabolism, and the increased levels of butyric acid mitigated CRC by increasing M1 TAMs polarization and suppressing M1 TAMs pyroptosis. In comparison to RF, PF demonstrates potential as a complementary therapeutic strategy in CRC, likely via modulation of butyrate metabolism and TAM pyroptosis.

## Supplementary Information

Additional file 1.

## Data Availability

Data will be provided on reasonable request.

## Abbreviations

AOM: Azoxymethane
CRC: Colorectal cancer
DAI: Disease activity index
DSS: Dextran sulfate sodium
EDC: 1-(3-Dimethylaminopropyl)-3-ethylcarbodiimide hydrochloride
FBS: Fetal bovine serum
GSDMD: Gasdermin D
IBD: Inflammatory bowel disease
IFN-γ: Interferon-γ
IL: Interleukin
ISTDs: Internal standards
LDA: Linear discriminant analysis
MRM: Multiple reaction monitoring
Nlrp3: NOD-like receptor 3
PBS: Phosphate buffer saline
PCA: Principal component analysis
PCR: Polymerase chain reaction
PF: Proessed frankincense
PF-H: PF high dose group
PF-M: PF medium dose group
PF-L: PF low dose group
PMA: Phorbol myristate acetate
PMSF: Phenylmethanesulfonyl fluoride
PVDF: Polyvinylidene fluoride
RF: Raw frankincense
RF-H: RF high dose group
RF-M: RF medium dose group
RF-L: RF low dose group
SDS-PAGE: Sodium dodecyl sulfate polyacrylamide gel electrophoresis
S/MCFAs: Short/medium chain fatty acids
TAM: Tumor-associated macrophage
UHPLC-TQ-MS: Ultra-high performance liquid chromatography triple quadrupole mass spectrometry
3NPH: 3-Nitrophenylhydrazine
