# Supplementary material for: Vinegar-processed frankincense extracts alleviate colorectal cancer by butyric acid mediating M1 tumor-associated macrophage pyroptosis
Source: Chin Med. 2025 Dec 1;20:208. doi: 10.1186/s13020-025-01260-5 (PMC12667045; doi:10.1186/s13020-025-01260-5)
Supplement: Supplementary file 1 — Additional file 1. [file 13020_2025_1260_MOESM1_ESM.docx]

Supplementary Material

# Supplementary Figures


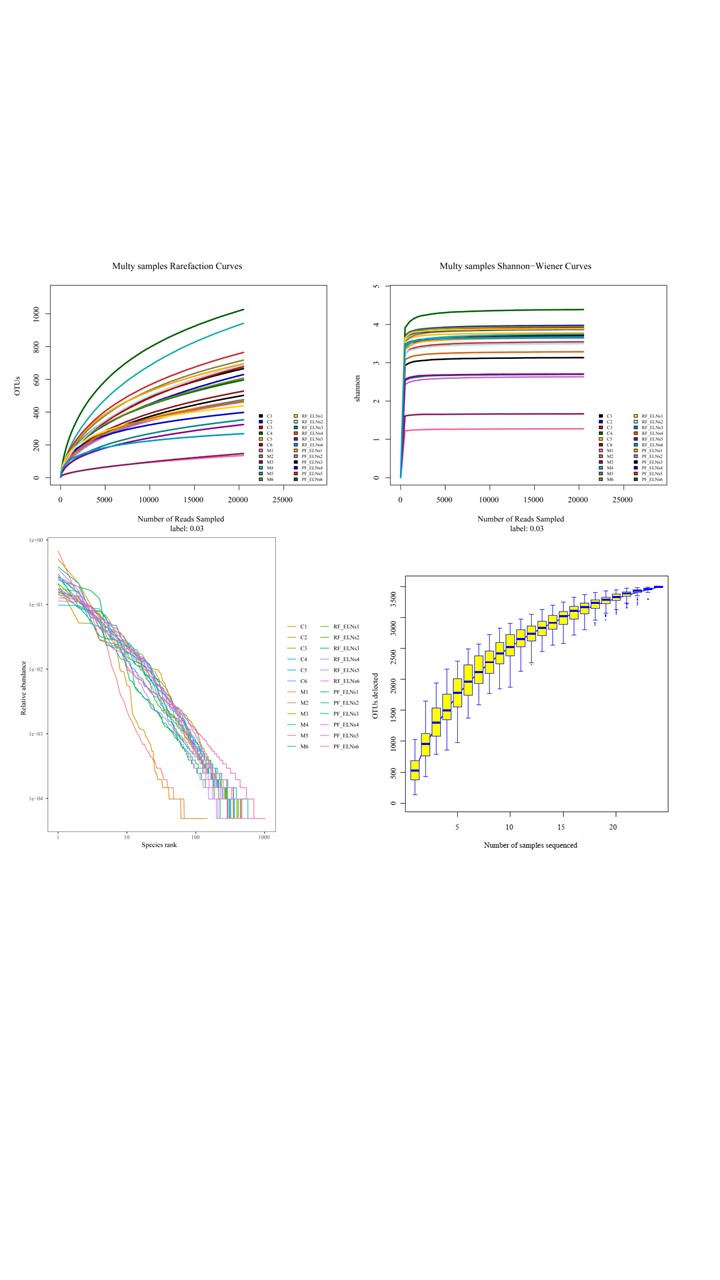


**Fig. S1** The rarefaction, shannon-wiener, rank-abundance and species accumulation curves for tested sample

# Supplementary Tables

**Table S1** The information of UHPLC-TQ-MS for S/MCFAs determination

| No. | Compound Name | Precursor Ion | Product Ion | Fragmentor | Collision Energy | RT | Polarity | ISTD |
| --- | --- | --- | --- | --- | --- | --- | --- | --- |
| 1 | Propionic acid | 208.4 | 208.4 | 100 | 0 | 10.842 | Negative | [^2^H_3_]-2-Methyl-propanoic acid |
| 2 | Butyric acid | 222.4 | 222.4 | 100 | 0 | 15.058 | Negative | [^2^H_3_]-2-Methyl-propanoic acid |
| 3 | Isobutyric acid | 222.4 | 222.4 | 100 | 0 | 14.5 | Negative | [^2^H_3_]-2-Methyl-propanoic acid |
| 4 | Valeric acid | 236.4 | 236.4 | 90 | 0 | 20.245 | Negative | [^2^H_2_]-Hexanoic acid |
| 5 | Isovaleric acid | 236.4 | 236.4 | 90 | 0 | 19.269 | Negative | [^2^H_2_]-Hexanoic acid |
| 6 | Caproic acid | 250.4 | 250.4 | 140 | 0 | 24.148 | Negative | [^2^H_2_]-Hexanoic acid |
| 7 | Enanthic acid | 264.4 | 264.4 | 150 | 0 | 26.705 | Negative | [^2^H_5_]-Heptanoic acid |
| 8 | Caprylic acid | 278.4 | 278.4 | 150 | 0 | 28.429 | Negative | [^2^H_5_]-Heptanoic acid |
| 9 | 2-Ethylhexanoic acid | 278.4 | 278.4 | 150 | 0 | 27.417 | Negative | [^2^H_5_]-Heptanoic acid |
| 10 | Pelargonic acid | 292.5 | 292.5 | 150 | 0 | 29.372 | Negative | [^2^H_3_]-Nonanoic acid |
| 11 | Carpic acid | 306.4 | 306.4 | 150 | 0 | 29.884 | Negative | [^2^H_3_]-Nonanoic acid |
| 12 | [^2^H_3_]-2-Methyl-propanoic acid | 225.4 | 225.4 | 100 | 0 | 14.393 | Negative | \ |
| 13 | [^2^H_2_]-Hexanoic acid | 252.4 | 252.4 | 150 | 0 | 24.112 | Negative | \ |
| 14 | [^2^H_5_]-Heptanoic acid | 269.4 | 269.4 | 150 | 0 | 26.585 | Negative | \ |
| 15 | [^2^H_3_]-Nonanoic acid | 295.4 | 295.4 | 150 | 0 | 29.326 | Negative | \ |

**Table S2** Primers for qPCR

| Gene name | Species | Forward sequence (5' to3') | Reverse sequence (5' to3') |
| --- | --- | --- | --- |
| *nlrp3* | mouse | GCTAAGAAGGACCAGCCACA | TCCCAGCAAACCTATCCACT |
| *caspase-1* | mouse | ATACAACCACTCGTACACGTCTTG | CAGATCCTCCAGCAGCAACTTC |
| *il-1β* | mouse | TCGCAGCAGCACATCAACAAG | TCCACGGGAAAGACACAGGTAG |
| *il-18* | mouse | AAATGACCAAGTTCTCTTCGTTGAC | CACAGCCAGTCCTCTTACTTCAC |
| *β-actin* | mouse | TCATCACTATTGGCAACGAGC | AACAGTCCGCCTAGAAGCAC |
| *NLRP3* | human | AGAGCCCCGTGAGTCCCATTAAG | CGCCCAGTCCAACATCATCTTCC |
| *CASPASE-1* | human | CACACCGCCCAGAGCACAAG | TCCCACAAATGCCTTCCCGAATAC |
| *IL-1β* | human | GACCTGGACCTCTGCCCTCTG | GCCTGCCTGAAGCCCTTGC |
| *IL-18* | human | GGCTGCTGAACCAGTAGAAGAC | AGCTTGCCAAAGTAATCTGATTCC |
| *β-ACTIN* | human | CACTCTTCCAGCCTTCCTTC | GTACAGGTCTTTGCGGATGT |
